# Cystic renal‐epithelial derived induced pluripotent stem cells from polycystic kidney disease patients

**DOI:** 10.1002/sctm.18-0283

**Published:** 2020-03-12

**Authors:** Annegien T. Kenter, Eveline Rentmeester, Job van Riet, Ruben Boers, Joachim Boers, Mehrnaz Ghazvini, Vanessa J. Xavier, Geert J.L.H. van Leenders, Paul C.M.S. Verhagen, Marjan E. van Til, Bert Eussen, Monique Losekoot, Annelies de Klein, Dorien J.M. Peters, Wilfred F.J. van IJcken, Harmen J.G. van de Werken, Robert Zietse, Ewout J. Hoorn, Gert Jansen, Joost H. Gribnau

**Affiliations:** ^1^ Department of Developmental Biology Erasmus Medical Center Rotterdam (EMC), Oncode Institute Rotterdam The Netherlands; ^2^ Department of Cell Biology Erasmus Medical Center Rotterdam (EMC) Rotterdam The Netherlands; ^3^ Department of Internal Medicine, Division of Nephrology and Transplantation Erasmus Medical Center Rotterdam (EMC) Rotterdam The Netherlands; ^4^ Cancer Computational Biology Center Erasmus Medical Center Rotterdam (EMC) Rotterdam The Netherlands; ^5^ Delft Diagnostic Laboratories (DDL) Rijswijk The Netherlands; ^6^ Department of Pathology Erasmus Medical Center Rotterdam (EMC) Rotterdam The Netherlands; ^7^ Department of Urology Erasmus Medical Center Rotterdam (EMC) Rotterdam The Netherlands; ^8^ Department of Clinical Genetics Erasmus Medical Center Rotterdam (EMC) Rotterdam The Netherlands; ^9^ Department of Clinical Genetics Leiden University Medical Center (LUMC) Leiden The Netherlands; ^10^ Department of Human Genetics Leiden University Medical Center (LUMC) Leiden The Netherlands; ^11^ Erasmus Center for Biomics Erasmus Medical Center Rotterdam (EMC) Rotterdam The Netherlands

**Keywords:** ADPKD, cyst, DNA methylation, epigenetic memory, iPS cells, PKD1, renal epithelial cells, second hit, somatic mutation

## Abstract

Autosomal‐dominant polycystic kidney disease (ADPKD) is the most common inherited kidney disease, leading to kidney failure in most patients. In approximately 85% of cases, the disease is caused by mutations in *PKD1*. How dysregulation of *PKD1* leads to cyst formation on a molecular level is unknown. Induced pluripotent stem cells (iPSCs) are a powerful tool for in vitro modeling of genetic disorders. Here, we established ADPKD patient‐specific iPSCs to study the function of *PKD1* in kidney development and cyst formation in vitro. Somatic mutations are proposed to be the initiating event of cyst formation, and therefore, iPSCs were derived from cystic renal epithelial cells rather than fibroblasts. Mutation analysis of the ADPKD iPSCs revealed germline mutations in *PKD1* but no additional somatic mutations in *PKD1/PKD2*. Although several somatic mutations in other genes implicated in ADPKD were identified in cystic renal epithelial cells, only few of these mutations were present in iPSCs, indicating a heterogeneous mutational landscape, and possibly in vitro cell selection before and during the reprogramming process. Whole‐genome DNA methylation analysis indicated that iPSCs derived from renal epithelial cells maintain a kidney‐specific DNA methylation memory. In addition, comparison of *PKD1*+/− and control iPSCs revealed differences in DNA methylation associated with the disease history. In conclusion, we generated and characterized iPSCs derived from cystic and healthy control renal epithelial cells, which can be used for in vitro modeling of kidney development in general and cystogenesis in particular.

## Graphical abstract

Autosomal dominant polycystic kidney disease (ADPKD) is the most common inherited kidney disease. Here, we generated induced pluripotent stem cells (iPSCs) of ADPKD patients to study kidney development and cyst formation in vitro. These iPSCs revealed germline and autosomal mutations implicated in ADPKD and displayed an epigenetic memory of kidney epithelial cells, providing powerful models to study ADPKD in vitro.

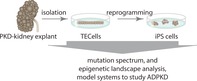

**Significance statement**

Autosomal dominant polycystic kidney disease (ADPKD) is the most common inherited kidney disease, leading to kidney failure in most patients. In approximately 85% of cases, the disease is caused by mutations in *PKD1*. How dysregulation of *PKD1* leads to cyst formation on a molecular level is unknown. The present study has generated induced pluripotent stem cells (iPSCs) of ADPKD patients to study the function of *PKD1* in kidney development and cyst formation in vitro. The iPSCs revealed germline and autosomal mutations implicated in ADPKD and displayed an epigenetic memory of kidney epithelial cells, providing powerful models to study ADPKD in vitro.

## 1 INTRODUCTION

Polycystic kidney disease (PKD) is a heterogeneous group of diseases that can be inherited or acquired. Autosomal dominant polycystic kidney disease (ADPKD) is the most common heritable form of PKD. Over time, these patients gradually acquire numerous cysts in both kidneys, resulting in renal function decline. Symptomatic treatment consists of blood pressure control, pain, and infection management. In addition, a vasopressin receptor antagonist (Tolvaptan) has become available, slowing renal decline in ADPKD patients with rapid progressing disease.^1^, ^2^, ^3^ However, most patients develop kidney failure and need a dialysis of a kidney transplantation before the age of 60.^4^

ADPKD is caused by a heterozygous germline mutation in *PKD1* (~85%), *PKD2* (~15%), or *GANAB* (~0.3%).^5^, ^6^, ^7^ *PKD1* encodes for polycystin‐1, a transmembrane protein, which structurally looks like a receptor or adhesion molecule and forms a complex with polycystin‐2, a calcium channel encoded by *PKD2*. GANAB, the alpha subunit of glucosidase II (GIIα), plays a role in glycosylation and quality control of polycystin‐1 in the endoplasmic reticulum.^7^ Expression of polycystin‐1 is high in the fetal kidney and essential for kidney development.^8^, ^9^ After nephron formation has completed, *PKD1* expression is reduced. In the adult kidney, the exact function of *PKD1* is unclear, but it is required in the renal epithelium to prevent cyst formation.

Cysts arise focally. The so‐called “second hit model” refers to the observation that all renal epithelial cells harbor a heterozygous mutation, but only a small proportion of the cells will form a cyst. In this model, somatic mutations affecting the remaining healthy *PKD1* allele are proposed to precede cyst initiation. This hypothesis is supported by the observation that heterozygous *Pkd1* mice develop only a few cyst, whereas (kidney specific) inducible knock out of both *Pkd1* alleles results in a severe cystic phenotype including renal failure, thus recapitulating the human phenotype.^10^ Further evidence supporting this second hit model came from mutational studies on DNA from cyst lining epithelium, isolated from human kidney tissue samples, which displayed small somatic mutations or loss of heterozygosity (LOH) in *PKD1* or *PKD2*.^11^, ^12^, ^13^, ^14^, ^15^ Moreover, the second hit might also be present in genes other than the one affected in the germline. Evidence for this *trans*‐heterozygous hypothesis is the identification of somatic mutations in *PKD2* in cyst DNA from patients with a *PKD1* germline mutation and vice versa.^15^, ^16^ Also copy number variations (CNVs) and small pathogenic somatic mutations at various loci in the genome of cyst lining cells have been reported.^17^, ^18^ However, the contribution of these mutations to cyst initiation has not been proven.

Conversely, there is also evidence against the second hit model. The second hit model does not explain cyst formation in autosomal recessive PKD, in which patients harbor a trans‐heterozygous mutation in *PKHD1*. Nor can it explain the rare patients who are trans‐heterozygous for an incompletely penetrant *PKD1* allele and a pathogenic *PKD1* allele.^19^ In these cases, patients already have both alleles mutated and still exhibit focal cyst formation. Moreover, *Pkd1*+/− mice develop cysts shortly after induction of renal injury, indicating *Pkd1* is haploinsufficient and a second hit in *Pkd1* is not required for cystogenesis.^20^ Finally, cystogenesis can also be provoked in normal kidneys—without a germline mutation in a PKD gene—by applying renal injury through drugs or ischemia.^21^, ^22^, ^23^, ^24^

Therefore, another mechanism for cyst formation has been proposed; the gene dosage model.^25^ This model hypothesizes that a variation in *PKD1* dosage is the underlying cause of cystogenesis. Reduction of *PKD1* expression levels could be the result of stochastic transcription fluctuations or inactivation of the *PKD1* gene by DNA methylation. Indeed, it was shown in mice that lowering *Pkd1* expression to approximately 10% of the original level results in a cystic phenotype.^19^, ^26^ Interestingly, also an increase in *Pkd1* expression was found to result in a cystic phenotype, confirming that regulation of proper *PKD1* levels is crucial.^27^, ^28^

In the last decade, induced pluripotent stem cells (iPSCs) have proven to be a powerful in vitro system for studying human genetic disorders.^29^, ^30^ The advantage of these iPSCs is their self‐renewing capacity, allowing indefinite expansion. This enables the use of a well‐characterized cell line for longer periods of time, reducing variation between experiments and allowing genome editing. Moreover, iPSCs are monoclonal. Importantly, recently developed protocols to differentiate iPSCs into kidney organoids make it a suitable system to study kidney development.^31^, ^32^, ^33^

Previously, iPSCs cells have been established from ADPKD patients heterozygous for a *PKD1* mutation.^34^, ^35^, ^36^, ^37^ Since these iPSCs were derived from fibroblasts, somatic mutations that might have contributed to cystogenesis will be missed. Second, several studies have shown that iPSCs retain an epigenetic signature of the tissue of origin.^38^, ^39^, ^40^ This residual epigenetic memory could contribute to a more efficient, directed differentiation back to the tissue of origin.^41^, ^42^ In this study, we established iPSCs derived from ADPKD patient cystic epithelial cells and from normal control kidney epithelial cells. Whole‐genome mutational analysis revealed heterozygous germline mutations in *PKD1* in all patients but no second hit in *PKD1* or *PKD2*. Genome‐wide DNA methylation analyses showed little differences between *PKD1*+/− and normal kidney‐derived iPSCs, but did reveal a kidney‐specific DNA methylation memory in renal epithelial derived iPSCs, not present in ESCs. These ADPKD iPSCs may provide a powerful model to study *PKD1* function and the involvement of the second hit in cyst formation and kidney development in vitro.

## 2 RESULTS

### 2.1 Generation and characterization of normal and cystic epithelial primary cells

**Figure 1:**
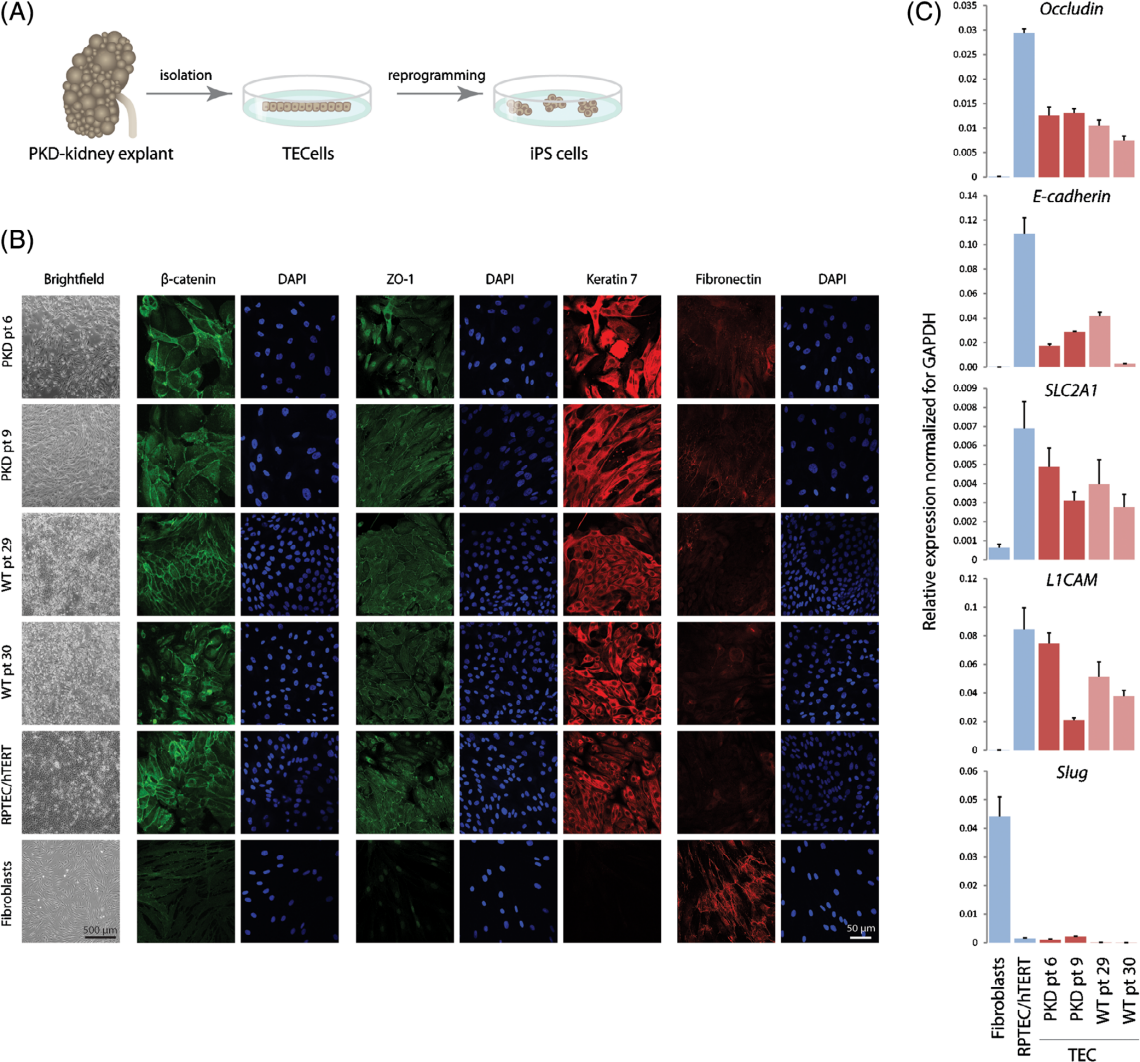
Generation and validation of normal and PKD‐patient derived tubular epithelial cells (TECs). A, Experimental setup: autosomal dominant polycystic kidney disease explants were used to isolate primary TECs, which were reprogrammed into induced pluripotent stem cells (iPSCs). B, Phase contrast microscopy and immunocytochemistry staining of junction markers ZO‐1 (tight junction) and β‐catenin (adherens junction), renal epithelial marker Keratin‐7, and mesenchymal marker fibronectin (scale bar = 50 μm for all panels). C, qRT‐PCR to determine expression of epithelial markers *OCLN/*Occludin (tight junction) and *CDH1*/E‐cadherin (adherens junction), renal tubular markers *SLC2A1* and *L1CAM*, and a mesenchymal marker *SNAI2/*Slug. RPTEC/hTERT cells and primary human fibroblasts were used as a positive and negative control, respectively. Ct values were normalized for GAPDH. The experiments were performed in triplicate twice; error bars represent the SD of both experiments

To generate human iPSC models, we established primary renal tubular epithelial cell (TEC) cultures from ADPKD kidney explants (Figure 1A). Each cell line was derived from a unique cyst, by using the inner epithelial monolayer of individual cysts. As controls, normal TECs were isolated from unaffected regions of kidneys that were resected because of a malignancy. In total, eight TEC lines were derived from two ADPKD patients and two normal individuals (Table 1). Both cyst‐derived as well as healthy control TECs displayed a typical epithelial morphology and no difference in karyotype stability (Figure 1B, Figure S1). To further confirm the epithelial origin of the TECs, we applied immunocytochemistry staining for epithelial junction markers (β‐catenin and ZO‐1), which showed an epithelial‐like honeycomb pattern, similar to an immortalized renal epithelial cell line (RPTEC/hTERT; Figure 1B). In addition, TECs were positive for KRT7, a renal epithelial marker, and negative for fibronectin, a mesenchymal marker, which is highly expressed in primary human fibroblasts (Figure 1B). These findings were supported by quantitative real‐time PCR (qRT‐PCR), revealing expression of epithelial junction markers (*OCLN*, Occludin and *CDH1*, E‐cadherin) and renal epithelial markers (*SLC2A1* and *L1CAM*) in all TEC cell lines (Figure 1C). In contrast, these cell lines did not express *SNAI2*/Slug, a mesenchymal marker (Figure 1C). These results confirm that the TEC lines are of epithelial origin.

**Table 1:** Patient characteristics

Patient number	Phenotype	Gender	Age	Germline mutation	Clinical features	TEC lines	iPSC lines
6	PKD	Male	58	*PKD1* c.11450delG/p.Gly3817fs (exon 41)	Infection	6.1/6.2/6.3	6.1A/6.1B
9	PKD	Male	45	*PKD1* c.4969delA/p.Arg1657fs (exon 15)	Space transplant	9.1/9.2/9.3	9.1A/9.1B
29	Healthy control	Male	41	NA	Tumor	29.1	29.1A/29.1B
30	Healthy control	Male	58	NA	Tumor	30.1	30.1A/30.1B

### 2.2 Cyst‐derived TECs harbor somatic mutations in various genes but not in *PKD1*

**Figure 2:**
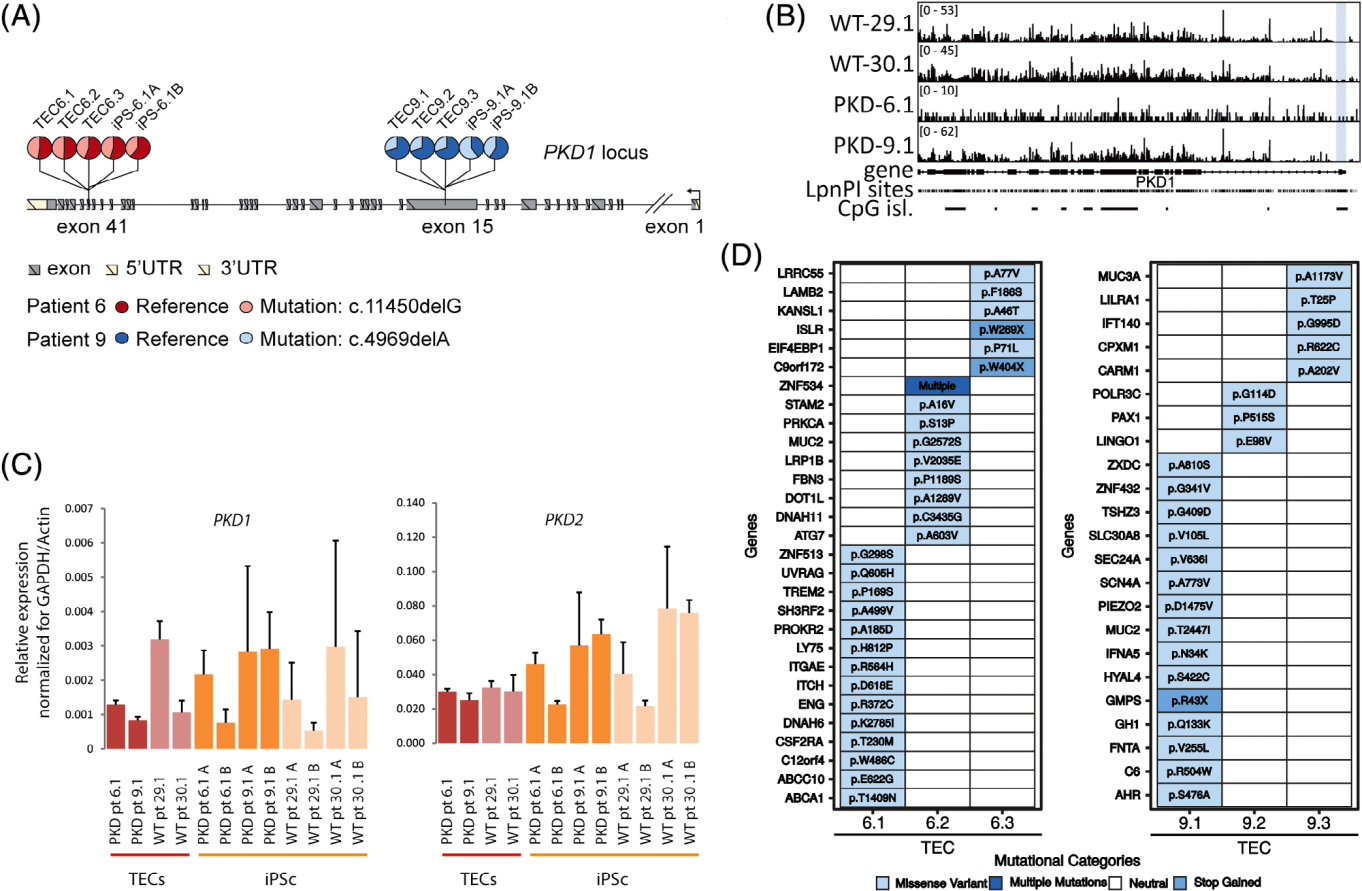
Germline and somatic mutation analysis cyst derived tubular epithelial cells (TECs). A, Heterozygous germline mutations in patient 6 and patient 9 present in TECs from 3 cysts result in a frameshift. B, MeD‐seq analysis of PKD1 showing read‐count scores per LpnPI site, revealing no increased DNA methylation in TECs obtained from cyst lining epithelium (promoter shown in blue). C, mRNA expression levels of *PKD1* and *PKD2* in TECs and iPSCs (qRT‐PCR), normalized by the average of two housekeeping genes; actin and GAPDH, error bars represent the SD. D, Somatic mutations observed by whole‐exome sequencing comparing cysts of the same patient

Both patients were diagnosed with ADPKD based on established clinical criteria.^43^ To investigate whether the patients carried a germline mutation in *PKD1* and to test if additional somatic mutations were present in *PKD1* or in other genes, we performed whole exome sequencing (WES) on TEC lines derived from three unique cysts, for each patient. We found a heterozygous, pathogenic (truncating/frame shift) mutation in *PKD1*, in exon 41 and 15 in patients 6 and 9, respectively (Figure 2A). We did not detect additional somatic mutations in *PKD1* in individual cyst‐derived TEC lines. However, because we could not exclude that small mutations (eg, single nucleotide variations and insertions/deletions or LOH in *PKD1* were missed in the WES data), we performed Long Range PCR (LR‐PCR) sequencing and multiplex ligation‐dependent probe amplification (MLPA) for *PKD1* specifically and found no somatic mutations in *PKD1* (data not shown). To test whether de novo DNA methylation was present at the remaining wild‐type allele of *PKD1*, which could lead to gene silencing, we applied MeD‐seq. This technique utilizes the methylation‐dependent restriction enzyme LpnPI to detect DNA methylation changes. MeD‐seq analysis did not reveal increased promoter methylation of the unaffected *PKD1* allele or changes in DNA methylation in the transcription start site (TSS, ± 1 kb), the gene body (starting 1 kb downstream of TSS until the transcription end sequence), as well as in gene proximal or distal regions (Figure 2B and data not shown), nor did we find increased DNA methylation of the *PKD2* or *PKHD1* alleles suggesting that these genes have not been affected by epigenetic silencing mechanisms (Figure S2A,B). To test whether the *PKD1* or *PKD2* mRNA expression level was affected in the ADPKD patient‐derived TECs, we performed qRT‐PCR, showing variation in expression level between samples, but no differences between ADPKD and normal TECs (Figure 2C). The lack of a second mutation in either *PKD1* or *PKD2* prompted us to test for the presence of other somatic mutations that might explain cyst formation. Somatic mutations were called through inter cyst comparisons (within each patient) only considering exonic regions and excluding synonymous mutations, identifying a total of 3 to 15 somatic mutations per cyst (Figure 2D). All mutations were heterozygous, or present in a fraction of the TEC cells, and except for *MUC2* were unique for one cyst. One cysts contained a pathogenic somatic mutation in *IFT140*, a ciliopathy gene that causes a cystic kidney phenotype,^44^ suggesting that this second hit could have had played a role in cyst initiation. For the other mutations, no established relationship with PKD has been reported yet. Thus, our analysis identified germline mutations in *PKD1* but no somatic mutations in *PKD1* or *PKD2*. Nonetheless, somatic mutations unique for individual cysts were found in various genes which may have contributed to cyst initiation in a trans‐heterozygous manner.

### 2.3 Cystic and normal renal epithelial cells can be reprogrammed to iPSCs

**Figure 3:**
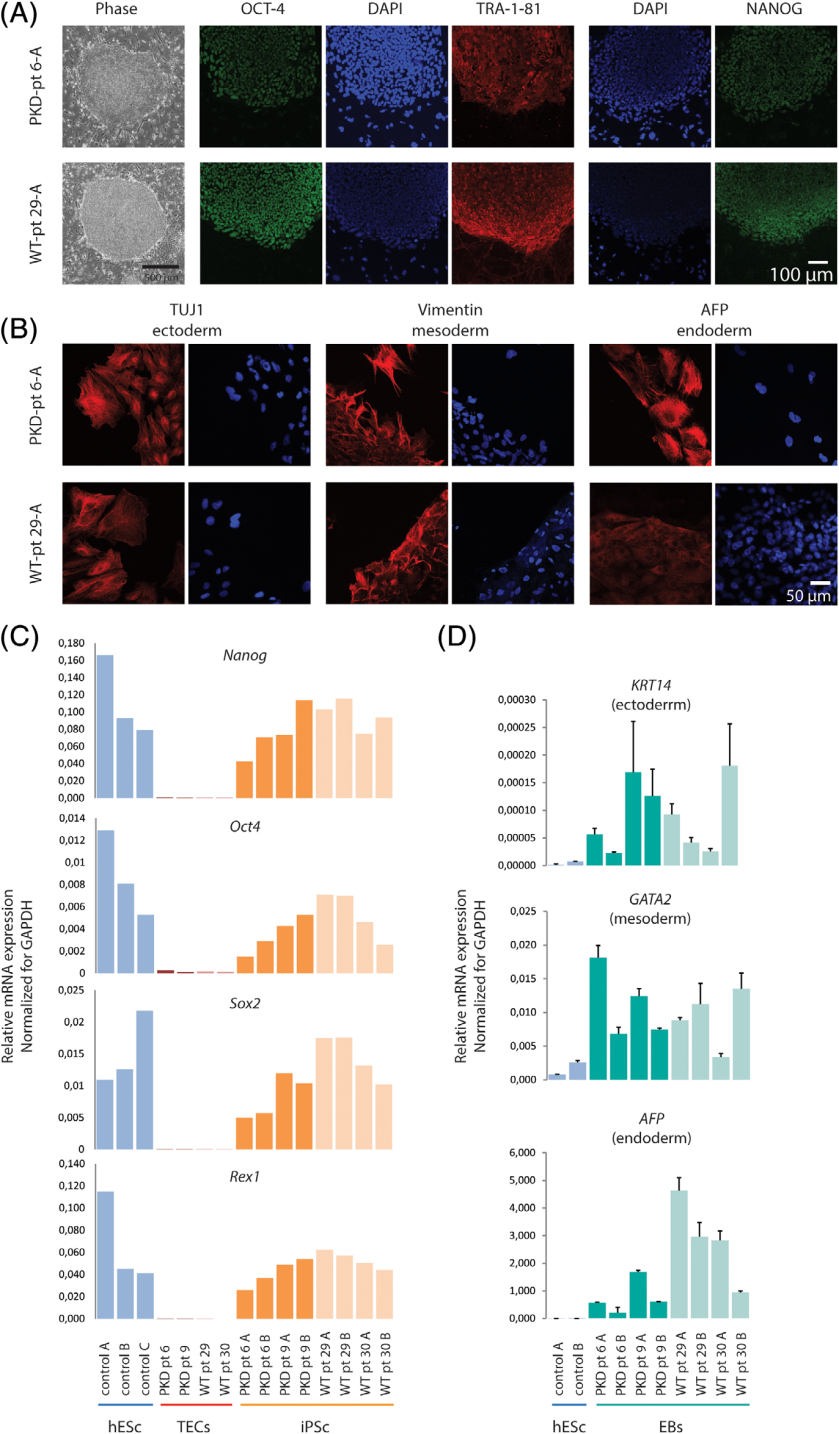
Establishment and characterization of polycystic kidney disease (PKD) patient and normal renal epithelial derived induced pluripotent stem cells (iPSCs). A, Bright field picture of morphology of representative PKD and wild‐type iPSC colonies. Shown are immunocytochemistry stainings for stem cell markers: OCT4, TRA‐1‐81, NANOG (scale bar = 100 μm for all panels). B, Random differentiation if iPSCs to embryoid bodies. Immunocytochemistry stainings for markers of all three germ layers: ectoderm (TUJ), mesoderm (Vimentin), endoderm (AFP) (scale bar = 50 μm for all panels). C, qRT‐PCR, detecting expression of endogenous pluripotency genes; *NANOG*, *OCT4*, *SOX2*, *and REX1*, iPSC lines and the parental tubular epithelial cell lines and positive control human embryonic stem cells (hESCs). D, Random differentiation of iPSCs to embryoid bodies. Expression of genes specific for each of the three germ layers is shown by qRT‐PCR; hESCs were used as negative control

Next, we established iPSCs of the TEC lines obtained from patients 6, 9, and controls. Of each primary TEC culture, one subclone was used to establish either patient‐derived‐cyst‐iPSC or control renal‐epithelium‐iPSCs. Early passage TECs were transduced with a polycistronic lentiviral vector expressing OCT4, SOX2, KLF4, and MYC, and a tdTomato reporter, under the control of a retroviral promoter (SFFV) that is rapidly silenced during the reprogramming process.^45^ Although an equal number of (TEC) cells was plated for transduction, cystic TECs were growing notably slower, resulting in a lower confluence at the time of transduction reducing iPSC colony formation efficiency. However, we did establish over 10 iPSC colonies for each of the original TEC lines. TdTomato‐negative iPSC colonies emerged from day 19 post‐transduction onward. Morphologically, no differences between PKD and normal iPSC colonies were observed. Two iPSC colonies from each TEC parental line were chosen for further characterization. These clones were grossly karyotypically normal (Figure S3A,B) and displayed expression of the nuclear stem cell markers, OCT4 and NANOG, and the stem cell surface marker TRA‐1‐81, determined by immunocytochemistry (Figure 3A and Figure S4). This was confirmed by qRT‐PCR indicating expression of the stem cells genes *NANOG*, *OCT4*, *SOX2*, and *REX1*, at levels comparable to the human embryonic stem cell (hESC), but not expressed in the parental TEC lines (Figure 3C). Embryoid body (EB) differentiation of iPSCs followed by immunocytochemistry staining detecting ectodermal (TUJ), mesodermal (Vimentin), and endodermal (AFP) marker gene expression indicated that the renal‐derived iPSCs possessed the capacity to differentiate in all three embryonic germ layers. This was confirmed at the RNA level by qRT‐PCR (Figure 3B,D and Figure S5). Our findings demonstrate that our renal epithelial derived iPSCs are genuine pluripotent stem cells.

### 2.4 Mutational and DNA methylation analysis of iPS cell lines

**Figure 4:**
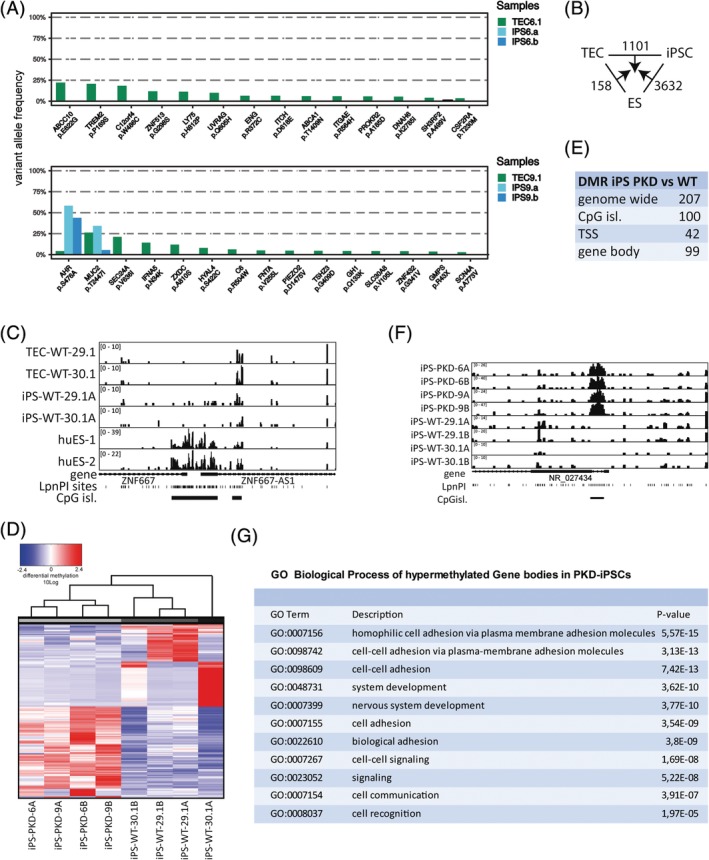
Inheritance of genetic and epigenetic polycystic kidney disease (PKD)‐associated modifications. A, Variant allele frequency of somatic mutations observed in tubular epithelial cell (TEC) lines 6.1 and 9.1, also observed in induced pluripotent stem (iPS) cell lines 6A/B and 9A/B, respectively, are shown. B, Total number of uniquely called differentially methylated regions (DMRs, TSS, CpG island, and gene body) excluding overlapping regions. C, MeD‐seq profiles for the *ZNF667* locus in wild‐type TEC, renal epithelium‐derived iPSC and embryonic stem cell lines. D, Unsupervised hierarchical clustering analysis of PKD and control iPSCs based on transcription start site DMRs observed between inter cell line comparisons. E, Overview of the number of DMRs observed in genome‐wide comparisons between PKD and control iPSC lines. F, MeD‐seq profiles showing a DMR observed between PKD and control iPSCs in an lncRNA gene. G, Gene Ontology (GO) analysis of genes hypermethylated DMRs in gene body region in PKD iPSCs

Each of the generated iPS cell lines represents an expanded single epithelial cell of the cyst. To investigate the mutational landscape in more detail, we performed WES on two iPS cell lines generated of TEC clones 6.1 and 9.1. To exclude de novo mutations potentially introduced during derivation of the iPS cell lines, we focused on mutations observed in the inter TEC comparisons described in Figure 2D. This analysis indicated that only two of the mutations observed in our TEC lines were present in all the iPSCs, indicating heterogeneity in the mutation spectrum in the TEC lines (Figure 4A).

Previous studies have indicated the presence of epigenetic memory of donor cells in the generated iPSCs. To test whether renal epithelial derived iPSCs retain renal epigenetic memory during iPS reprogramming, which might be beneficial for iPSC differentiation toward the renal lineage, we applied MeD‐seq on DNA isolated from undifferentiated iPSC clones, hESCs, and TEC lines. Differentially methylated regions (DMRs) between three predetermined groups, six iPSC lines (PKD patient and controls), five control ESCs, and four renal epithelial cell lines were detected genome‐wide using a sliding window approach. Statistically significant regions with a more than twofold difference in read count were selected. This analysis revealed that our iPSCs were properly reprogrammed showing demethylation of the HoxA locus and loci involved in pluripotency (Figure S6A and data not shown). Hierarchical clustering based on genome‐wide DMRs detected between TEC‐iPS‐hES indicated that iPS cell lines derived from kidney epithelium cluster away from hES cell lines except huES8, suggesting the presence of a kidney epithelial epigenetic signature (Figure S6B). Indeed, inter TEC‐iPS‐ES comparisons indicated many more TSS, CpG island. and gene body‐associated DMRs that were unique to ES than to iPS cells indicative for the presence of an epigenetic heritage of TECs (Figure 4B,C and Figure S6C).

Next, we compared cyst‐derived and control renal‐epithelial iPS cells to test whether we could detect differences in DNA methylation associated with disease history. Hierarchical cluster analysis based on regions that were differentially methylated between PKD and control samples clustered PKD iPS cells away from WT iPS cells (Figure 4D). Genome‐wide determination of DMRs using a sliding window approach revealed 207 DMRs between PKD and control iPSCs (Figure 4E,F). Gene ontology (GO) analysis with PKD‐specific significantly hypermethylated gene body DMRs did retrieve GO terms like cell‐cell adhesion and cell‐cell signaling, processes which are implicated to be disturbed in PKD (Figure 4G). These results indicate that renal iPSCs are reprogrammed into pluripotent iPSCs but display an epigenetic signature of the TECs they have been derived of, providing powerful models to study PKD disease by in vitro differentiation.

## 3 DISCUSSION

Here, we have generated iPSCs from cystic renal epithelial cells. iPSCs from healthy renal epithelial cells have been established previously,^46^, ^47^ but this was not yet done from cyst cells. We found that our cyst‐iPSCs contain somatic mutations, but only a few of these mutations were also present in TECs they were derived from. In addition, we show that these renal‐iPSCs retain residual epigenetic kidney memory, which can be beneficial in directed differentiation to kidney organoids.

Using WES and additional mutation analysis of *PKD1* specifically (LR‐PCR and MLPA), we found that both ADPKD patients have germline mutations in *PKD1*, but we did not find somatic mutations in or LOH of *PKD1* in TECs derived from cysts of these patients. In addition, we did not detect reduced *PKD1* mRNA levels. Off note, we also did not detect increased methylation in the promotors of *PKD1*, *PKD2*, or *PKHD1*, suggesting that epigenetic silencing of these genes did not lead to cystogenesis. The fact that we did not find genetic mutations nor epigenetic alterations in *PKD1* could mean that a second hit did not occur in *PKD1 or PKD2*, but that merely the germline mutation leads to haploinsufficiency which is in line with previous findings that *Pkd1*+/− mice develop a cystic phenotype when renal injury is induced.^20^ Alternatively, a second hit in *PKD1* may have occurred in the cyst but was lost during culture of the primary TECs used in this study. This could be explained by polyclonality of the cysts, as reported previously,^48^ or a growth advantage in cell culture of cells with a single germline mutation over cells that were *PKD1* null, as were reported for other systems, were cancer cells, or were outgrown by wild‐type cells in standard culture conditions.^49^

Remarkably, we found several somatic mutations in genes other than *PKD1 or PKD2*, ranging from 3 to 15 mutations per cyst. This is in line with a recent study showing that cyst cells contain somatic mutations in non‐*PKD1/PKD2*, ciliopathy, or cancer‐related genes.^18^ In concordance with that, many of the genes affected by a somatic mutation identified in our analysis are also linked to the cilium or cancer. Moreover, in one TEC line, a somatic mutation was found in *IFT140*, a gene that causes a cystic kidney phenotype in mice.^44^ In addition, some of the affected genes are known to function in pathways previously linked to cystogenesis; *ITCH*, as a negative regulator of Wnt signaling like *PKD1* itself,^50^ and *ENG*, being a component of the transforming growth factor beta receptor complex, also a pathway implicated in PKD.^51^, ^52^ Finally, *MUC2* is a family member of the mucin *MUC1*, a gene causing autosomal dominant tubulointerstitial kidney disease.^53^ Somatic mutations in non‐*PKD1/PKD2* genes found by us and others could be merely bystander mutations due to local damage. This may explain the relative low variant allele frequency that we observed as a representation of a heterogeneous population cells with different mutations and is in line with our findings that only a few mutations are found back in reprogrammed iPS cell lines. Alternatively, these mutations might have played a role in cyst initiation, but an ex vivo growth advantage of unaffected cells might have diminished the variant allele frequency.

Several previous reports have shown that residual DNA‐methylation provides a transcriptional memory and favors directed differentiation back to the lineage of origin.^40^, ^41^, ^42^ When comparing the DNA methylation profiles of human ESCs, renal derived iPSCs, and the parental renal TEC lines, we found many more ESC than iPS unique DMRs, indicating the presence of an epigenetic memory in reprogrammed iPSCs. Whether this memory is related to the reprogramming process itself or to the cell type of origin needs further investigation including MeD‐seq analysis on reprogrammed fibroblasts. Nevertheless, iPSCs generated from PKD cyst epithelial cells did contain many DMRs when compared with iPS cells generated from control material, suggesting that at least some of the epigenetic heritage is kidney epithelial specific. GO analysis of genes displaying gene body hypermethylation, identifying active genes or genes with an active history, revealed these genes to function in cell–cell interaction and cell orientation, suggesting an (renal) epithelial DNA methylation profile representative for the disease history. We therefore conclude that iPS cell lines derived from kidney epithelial cell lines display a kidney epithelial as well as disease‐specific epigenetic memory. R‐iPSCs may therefore represent better models to differentiate to kidney organoids in terms of differentiation efficiency and resemblance of the in vitro organoids to the actual kidney.

## 4 METHODS

### 4.1 Sample collection and TEC culture

Polycystic kidney explants were obtained from patients diagnosed with ADPKD based on radiological imaging.^43^ Collection was approved by the Medical Ethics Committee of the Erasmus Medical Center (MEC20130‐188). TEC culture isolation protocol was adapted from Klinkel et al^54^ and performed as follows: samples were immediately placed on ice. Membranous layers were aseptically removed from the kidney, and cyst, which were filled with clear fluid, were carefully dissected. The inner epithelial layer of cysts were separated manually from the cyst wall, washed with phosphate‐buffered saline (PBS), and cut into small fragments of approximately 1 mm^2^. Normal control kidney samples were also washed in PBS and cut into fragments of the same size. Next, the kidney fragments were plated on 0.2% gelatin‐coated 10‐cm plates and incubated until fragments had adhered to the plate after which the TEC medium was carefully added; TEC‐medium: DMEM:Ham's F12 (1:1) media (Gibco life), supplemented with 100 U/mL penicillin‐streptomycin, 100× Insulin‐Transferrin‐Selenium (Thermo Fisher Scientific), 40 pm triiodo‐l‐thyronine (Sigma), 36 ng/mL hydrocortisone (Sigma), 10 ng/mL recombinant human EGF (Peprotech). Medium was refreshed two to three times a week, and cells were passaged by trypsinization when reaching 80% confluence.

### 4.2 TEC reprogramming into iPSC with lentiviral vector

TEC with a low passage number (p4‐p5) were used for reprogramming. For each TEC line, a total of 2 × 10^5^ cells/well were plated in a six‐well culture plate coated with 0.2% gelatin. The following day, TECs were transduced with a lentivirus encoding *OCT*, *SOX2*, *KLF4*, *c‐MYC*.^45^ To increase the efficiency of viral transduction, 4 μg/mL of polybrene (Invitrogen) was added. Day 4 post‐transduction, cells were replated on gamma‐irradiated mouse embryonic fibroblasts (MEFs). The next day, the media was converted to hESC media (DMEM/F12, 20% knock‐out serum, 1% l‐glutamine, 1% nonessential amino acids, 0.1 mM β‐mercaptoethanol, 10 ng/mL basic fibroblast growth factor [bFGF]). Between days 2 and 9, 2 mmol/L of valproic acid was added daily. Around day 26 onward, the iPSC colonies were picked and expanded.

### 4.3 Karyotype analysis

iPSCs were cultured in feeder‐free conditions on Geltrex coating (Gibco, A1413301) and in E8 medium (Gibco, Cat A14666SA). Cells were harvest using TrypLE Express Enzyme (Gibco, LS12604021) and collected in a 15‐mL tube with E8 medium supplemented with (10 μg/mL) Colcemid (Gibco 15 210‐040) and incubated at 37°C for 30 minutes. Next, cells were treated with 0.075 M KCl for 10 minutes at 37°C and another 10 minutes at room temperature (RT). Cells were fixed with fresh Carny's fixative solution **(**3:1 methanol:acetic acid), streaked onto glass slides and stained with Vectashield Mounting with DAPI (Vector Laboratories, H‐1200). At least 10 metaphases were analyzed per iPSC line. For karyotype analysis of TECs, cells were cultured in TEC medium and treated with (12 μg/mL) Colcemid (Gibco 15 210‐040) at 37°C for 6 hours. Next, cells were harvest using TrypLE Express Enzyme and processed with the HANABI chromosome harvester (ADS Biotec) and stained with Quinacrine. To map the deletions in our iPSC lines, SNP array analysis was performed using Human CYTO SNP 12 version 1 arrays (Illumina, San Diego, California), aligned to human genome build 19.

### 4.4 In vitro differentiation of iPSCs to EBs

To induce EB formation, iPSCs were dissociated from the MEF feeder layer using collagenase IV 1 mg/mL, harvested by centrifugation at 200*g* for 2 minutes and cultured on ultralow attachment six‐well plates (Corning) in hESC medium without bFGF. Medium was refreshed every other day. Day 8 EBs were collected for RNA analysis. For immunocytochemistry analysis, Day 8 EBs were seeded on Nunc Lab‐Tek chamber slide to attach and grow till day 16.^55^

### 4.5 Immunocytochemistry iPSC/TEC + microscopy

iPS cells were fixed for 15 minutes with 4% paraformaldehyde at RT followed by permeabilization of the cells using 0.1% Triton X‐100 (PBS) for 10 minutes. After blocking (1% BSA, 0.05% Tween 20 in PBS) for 30 minutes, cells were stained for 1 hour at RT or overnight at 4°C with primary antibodies, followed by incubation with secondary antibodies. Antibodies are listed in the Appendix (Table 1). Images were acquired with a Leica SP5 confocal microscope and processed using Fiji.

### 4.6 DNA isolation

Cells were collected by centrifugation after collagenase treatment and lysed overnight at 37°C in lysis buffer (0.2% sodium dodecyl sulfate and 1 mg/mL Proteinase K). The next day, phenol and chloroform extractions were performed and DNA was precipitated using isopropanol and washed with 70% ethanol. Finally, DNA was dissolved in 10 mM Tris buffer (pH 7.5).

### 4.7 Whole exome sequencing and analysis

Genomic DNA (gDNA) was collected from TECs at passage <5, DNA was sheared in a Covaris S220 instrument, and prepared for sequencing using SureSelectXT reagents and Clinical Research Exome capture baits (Agilent Technologies). For the low‐input samples (where less than 1 μg genomic DNA was available), we used 200 ng input gDNA and the manual sampleprep protocol provided by the manufacturer. For the remaining samples, we used the automated sampleprep protocol on an Agilent Bravo B system and up to 3 μg input gDNA. Sequencing was done either on an Illumina HiSe2500 or HiSeq4000 system, for paired‐end 100 or 150, respectively. At least 5.2 Gbp of sequencing data per sample was generated. Sequence reads were mapped against human reference genome GRCh38 using Burrows‐Wheeler Aligner (v0.7.12) with default settings and supplying respective read‐groups.^56^ In the case of iPSC samples, sequence reads were mapped against both the human reference genome GRCh38 and mouse reference genome GRCm38 using Burrows‐Wheeler Aligner (v0.7.16a) with default settings. Afterwards, reads originating from mouse were discarded by Disambiguate (v1.0.0).^57^ After alignment and quality control, sequence reads originating from multiple lanes were merged using GATK PrintReads (v3.6.0) prior to further analysis.^58^ Sequence duplicates were marked using PicardTools (v1.129).^59^ Somatic and germline variant calling was performed by Strelka2 (v2.8.3) using a matched‐normal design with default exome settings.^60^ In the absence of matched normal material (patients 6 and 9), randomized alternative cyst samples of the same patient were used as substitute “matched‐normal” reference. Variants were annotated with GENCODE annotations using ANNOVAR.^61^, ^62^ Heuristic filtering removed variants, which did not pass all standard Strelka2 post‐calling filters, had fewer than six total reads or had an allelic frequency above 0.02% in the ExAC population.^63^ CONTROL‐FREEC (v11.0) was used to detect copy‐number aberrations using the same matched normal scheme as previously described with default exome settings on SureSelect v5 target regions.^64^ Genomic data were visualized with the R statistical platform using the TrackViewer and RCircos BioConductor packages.^65^, ^66^

### 4.8 LR‐PCR‐sequencing and MLPA of PKD1

For the repeated region of PKD1 (exon 1‐33), an LR‐PCR was used followed by a nested PCR while the unique part (exon 33‐46) and the complete coding region of PKD2 was directly amplified. PCR products were Sanger‐sequenced using standard procedures (primers sequences and conditions available upon request). For the detection of larger deletions and duplications, two commercially available MLPA kits (P351‐B2 and P352‐C1; MRC‐Holland, Amsterdam, The Netherlands) were used according to the manufacturer's instructions.

### 4.9 RNA isolation, cDNA synthesis, and quantitative real‐time PCR

TECs were lysed at passage < p5 in Tri reagent (Sigma) for 5 minutes. After chloroform extraction, RNA was precipitated using isopropanol and washed with 75% ethanol. RNA was dissolved in 20 μL DepC‐treated H_2_0 and stored at −80°C. To remove DNA, RNA samples were incubated with 1 U DNase (Thermo Fisher Scientific) for 30 minutes at 37°C. DNAse was stopped by incubating with EDTA (25 mM) at 65°C for 10 minutes. Random hexamers (stock 50 μM, final 5 μM, Thermo Fisher Scientific) and dNTPs (10 mM) were added and incubated 65°C for 5 minutes. After denaturation, samples were placed on ice and RT mix was added containing 5× first strand buffer, DTT (0.1 M), and RNase out (company). Samples were incubated at 25°C for 2 minutes. Next 200 U Superscript II was added (Thermo Fisher Scientific) and incubated for 10 minutes at 25°C and 15 minutes at 70°C. Samples were stored at −20°C. Quantitative real‐time PCR was performed in a 10‐μL final reaction volume using Platinum Taq DNA polymerase (Life Technologies) and Sybr Green (Sigma Aldrich) in a CFX 384 Real Time system (BioRad). Expression levels were normalized to Actin/GAPDH. Primer sequences are listed in Appendix (Table 2).

### 4.10 MeD‐seq sample preparation

DNA from iPSC samples collected at passage 12 were used for MeD‐seq analysis. LpnPI and MspJI (New England Biolabs) digestions were carried out according to the manufacturer's protocol. Reactions contained 50 ng in a 10‐μL volume and digestion took place overnight in the absence of enzyme activators. Digests of genomic DNA with LpnPI resulted in snippets of 32 bp around the fully methylated recognition site that contains CpG. The DNA concentration was determined by the Quant‐iT High‐Sensitivity assay (Life Technologies; Q33120) and 50 ng ds DNA was prepared using the ThruPlex DNA‐seq 96D kit (Rubicon Genomics cat#R400407). Twenty microliters of amplified end product was purified on a Pippin HT system with 3% agarose gel cassettes (Sage Science). Stem‐loop adapters were blunt‐end ligated to repaired input DNA and amplified (4 + 10 cycles) to include dual‐indexed barcodes using a high fidelity polymerase to yield an indexed Illumina NGS library. Multiplexed samples were sequenced on Illumina HiSeq2500 systems for single read of 50 base pairs according to the manufacturer's instructions. Dual‐indexed samples were demultiplexed using the bcl2fastq software (Illumina).

### 4.11 MeD‐seq data processing

Data processing was carried out using specifically created scripts in Python version 2.7.5. Raw fastq files were subjected to Illumina adaptor trimming, mouse genome‐specific reads were removed, and reads were filtered based on LpnPI restriction site occurrence between 13 and 17 bp from either 5′ or 3′ end of the read. Reads that passed the filter were mapped to hg38 using bowtie2.1.0. Multiple and unique mapped reads were used to assign read count scores to each individual LpnPI site in the hg38 genome. SAM and BAM files were generated using SAMtools for visualization. Gene and CpG island annotations were downloaded from UCSC (HG38). Genome‐wide individual LpnPI site scores were used to generate read count scores for the following annotated regions: TSS (1 kb before and 1 kb after), CpG islands and gene body (1 kb after TSS till TES).

### 4.12 MeD‐seq data analysis

Data analysis was carried out in Python version 2.7.5. DMR detection was performed between two data sets containing the regions of interest (TSS, gene body or CpG islands) using the chi‐squared test on read counts. Significance was called by either Bonferroni or FDR using the Benjamini‐Hochberg procedure. DMRs were used for unsupervised hierarchical clustering (complete/city‐block); the *Z*‐score of the read counts was used for normalization and is also shown in the heatmaps. In addition, a genome‐wide sliding window was used to detect sequentially differentially methylated LpnPI sites. Statistical significance was called between LpnPI sites in predetermined groups using the chi‐squared test. Neighboring significantly called LpnPI sites were binned and reported, DMR threshold was set at a minimum of 10 LpnPI sites, a minimum size of 100 bp, and either a twofold or fivefold change in read counts. Overlap of genome‐wide detected DMRs was reported for TSS, CpG island, and gene body regions. GO analysis was performed in Gorilla (FDR‐adjusted).

## CONFLICT OF INTEREST

The authors declare no potential conflicts of interest.

## AUTHOR CONTRIBUTIONS

A.T.K.: conceived and planned the experiments, carried out the experiments, collected patient material, performed the data analysis, wrote the manuscript; R.Z., E.H., G.J.: conceived and planned the experiments, wrote the manuscript; J.G.: conceived and planned the experiments, performed the data analysis, wrote the manuscript; E.R., J.B., M.G., V.X., R.B. M.T., B.E., M.L., D.P., W.I.J.: carried out the experiments; G.L., P.V.: carried out the experiments, collected patient material; A.K.: carried out the experiments, performed the data analysis; J.R., H.W., B.E.: performed the data analysis. All authors discussed the results and contributed to and approved the final manuscript.

## Supporting information


**Appendix** **S1**: Supplementary methods

**Figure S1** Karyotype of TEC lines.
(A) Quantification and (B) graphic representation of karyotyping of TEC lines 6.1, 6.2, 9.1 and 9.2. Cells with an abnormal karyotype only showed loss of chromosomes, with no preference for loss of one specific chromosome.

**Figure S2** MeD‐seq analysis of PKD2 and PKHD1 loci in WT and PKD TECs.
MeD‐seq profiles of (A) *PKD2*, and (B) *PKHD1*, displaying read‐counts per LpnPI site, showing no gain in methylation in the promoter (blue), or other regions of both genes.

**Figure S3** Karyotype of iPSC lines.
(A) Quantification of karyotyping of iPSC lines, cells with an abnormal karyotype only showed loss of chromosomes, with no preference for loss of one specific chromosome. (B) SNP Array analysis per clone showing Log(R ratio) potentially detecting gains and losses (top panels per clone), and loss of heterozygosity with B allele frequency (BAF) of 100% or 0% (bottom panels per clone).

**Figure S4** Characterization of undifferentiated WT and PKD derived iPSC lines.
Immuno‐fluorescence expression analysis of pluripotency markers OCT4 (FITC), TRA‐1‐81 (Rhodamine Red) and NANOG (FITC, DNA is DAPI/Blue) in iPSC lines derived from PKD and WT TEC cell lines.

**Figure S5** EB differentiation and germ layer formation of PKD and WT iPSC lines.
Immuno‐fluorescence expression analysis of endoderm, mesoderm and ectoderm markers AFP, Vimentin, and TUJ1 (Rhodamine red, DNA is DAPI/Blue) in EB differentiated iPSC lines derived from PKD and WT TEC cell lines (scale bar = 50 μm for all panels).

**Figure S6** Genes hypermethylated in gene body in PKD iPSCs.
(A) MeD‐seq gene tracks of the HOXC locus in TEC, iPS and ES cell lines, showing loss of methylation in reprogramed iPS‐6A and iPS‐9A cell lines to a level similar to found in ESCs. (B) Unsupervised hierarchical clustering analysis of TEC, PKD iPS and control ES cell lines, based on TSS DMRs observed between inter cell line comparisons. (C) Overview of CpG island, TSS and gene body DMRs specific for TEC vs iPSC:ESC, iPSC vs ESC:TEC, and ESC vs TEC:iPSC comparisons.

## Data Availability

The data that support the findings of this study are available from the corresponding author upon reasonable request. The WES and MeD‐seq data from this study have been submitted to the Gene Expression Omnibus^67^ database under the accession number PRJNA600136, except HuES‐8, which is available under PRJNA375757 as these data were already published.
