# Supplementary material for: Cystic renal‐epithelial derived induced pluripotent stem cells from polycystic kidney disease patients
Source: Stem Cells Transl Med. 2020 Mar 12;9(4):478–90. doi: 10.1002/sctm.18-0283 (PMC7103626; doi:10.1002/sctm.18-0283)
Supplement: Supplementary file 1 — Appendix S1: Supplementary methods [file SCT3-9-478-s001.docx]

**Supplementary methods**

**Table 1 Antibodies**

|  | Antibody | Dilution | Company |
| --- | --- | --- | --- |
| Embryonic Germ layer markers | Mouse anti-Alpha Fetoprotein / AFP | 1:100 | R&D Systems  Cat# MAB1368 |
|  | Mouse anti-β-Tubulin III / TUJ-1 | 1:500 | Sigma  Cat# T8660 |
|  | Mouse anti-Vimentin | 1:100 | Santa Cruz  Cat# SC-6260 |
| Pluripotency  markers | Mouse anti-TRA-1-81 | 1:200 | BD Pharmingen  Cat# 560072 |
|  | Rabbit anti-OCT4 | 1:250 | Abcam  Cat# AB19857 |
|  | Rabbit anti-Nanog | 1:100 | Abcam  Cat# AB21624 |
| TEC  markers | Rabbit anti-Beta-catenin | 1:100 | Santa Cruz  Cat# Sc-7199 |
|  | Rabbit anti-ZO-1 | 1:100 | Invitrogen  Cat# 40-2200 |
|  | Mouse anti-Keratin-7 | 1:100 | GenTex  Cat# GTX40206 |
|  | Mouse anti-Fibronectin | 1:100 | Sigma  Cat# F6140 |
| Secondary | Goat anti-Mouse IgM (H+L) Alexa Fluor 546 | 1:500 | Invitrogen  Cat# A-21045 |
|  | Goat anti-Mouse IgG (H+L) Alexa Fluor 546 | 1:500 | Invitrogen  Cat# A-11003 |
|  | Goat anti-Mouse IgG (H+L) Dylight 594 | 1:400 | Jackson Immuno Research  Cat# 115-585-146 |
|  | Goat anti-Rabbit IgG (H+L) Alexa Fluor 488 | 1:400 | Invitrogen  Cat# A-11008 |

**Table 2 qRT-primers**

| Primer | F/R | Sequence 5’to 3’ |
| --- | --- | --- |
| Nanog | F | CAG CCC CGA TTC TTC CAC CAG TCC C |
|  | R | CGG AAG ATT CCC AGT CGG GTT CAC C |
| OCT3/4 | F | GAC AGG GGG AGG GGA GGA GCT AGG |
|  | R | CTT CCC TCC AAC CAG TTG CCC CAA AC |
| Sox2 | F | GGG AAA TGG GAG GGG TGC AAA AGA GG |
|  | R | TTG CGT GAG TGT GGA TGG GAT TGG TG |
| Rex1 | F | CAG ATC CTA AAC AGC TCG CAG AAT |
|  | R | GCG TAC GCA AAT TAA AGT CCA GA |
| KRT14 | F | CAC CTC TCC TCC TCC CAG TT |
|  | R | ATG ACC TTG GTG CGG ATT T |
| GATA2 | F | TGA CTT CTC CTG CAT GCA CT |
|  | R | AGC CGG CAC CTG TTG TGC AA |
| AFP | F | GAA ACC CAC TGG AGA TGA ACA |
|  | R | CTG CAG CAG TCT GAA TGT CC |
| Occludin | F | TGC CGC GTT GGT GAT CTT T |
|  | R | GCC CAG GAT AGC ACT CAC TAT T |
| E-cadherin | F | CCC ACC ACG TAC AAG GGT C |
|  | R | ATG CCA TCG TTG TTC ACT GGA |
| SLC2A1 | F | CTT TTC TGT TGG GGG CATGAT |
|  | R | CCG CAG TAC ACA CCG ATG AT |
| L1CAM | F | CAA GCC CGA AGT GCA GTT C |
|  | R | CTG GCA AAG CAG CGG TAG AT |
| Slug | F | ATA TTC GGA CCC ACA CAT TAC CT |
|  | R | GCA AAT GCT CTG TTG CAG TGA |
| PKD1 | F | AAG GAA GAA GCC CGC AAG |
|  | R | CAT GAG GCA TCC CCA TAG C |
| PKD2 | F | ACC GTG GAT GAC ATT TCA GA |
|  | R | TGC CTC AAT CTC TGC ATC AGT |
| Actin | F | GTC TTC CCC TCC ATC GTG |
|  | R | AGG GTG AGG ATG CCT CTC TT |
| GAPDH | F | GGT GGT GCA GGA GGC ATT |
|  | R | GCC AAA AGG GTC ATC ATC TC |
