# Supplementary figures and images for: Cystic renal‐epithelial derived induced pluripotent stem cells from polycystic kidney disease patients
Source: Stem Cells Transl Med. 2020 Mar 12;9(4):478–90. doi: 10.1002/sctm.18-0283 (PMC7103626; doi:10.1002/sctm.18-0283)

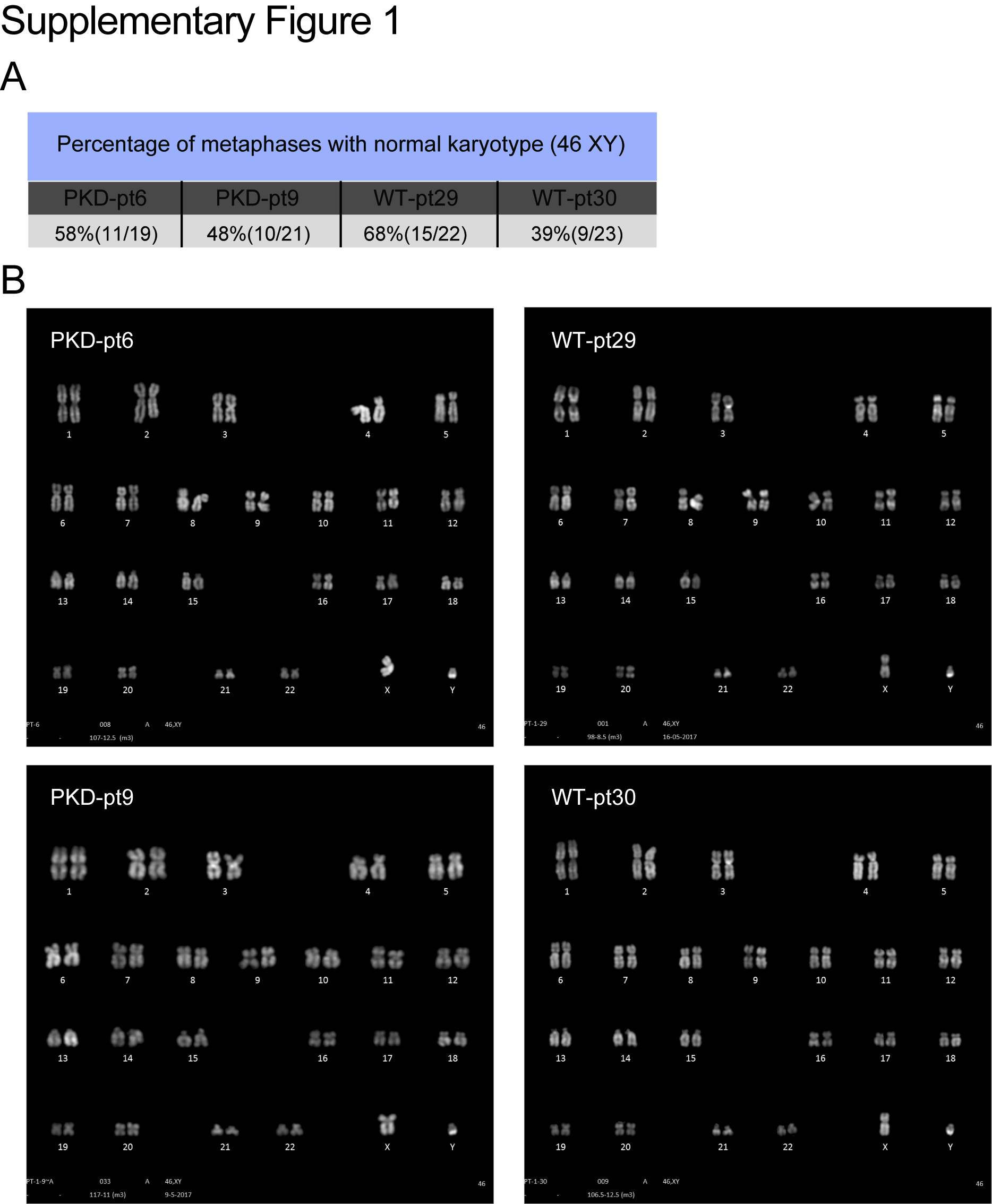

Supplement: Supplementary file 2 — Figure S1 Karyotype of TEC lines. (A) Quantification and (B) graphic representation of karyotyping of TEC lines 6.1, 6.2, 9.1 and 9.2. Cells with an abnormal karyotype only showed loss of chromosomes, with no preference for loss of one specific chromosome. [file SCT3-9-478-s002.tif]

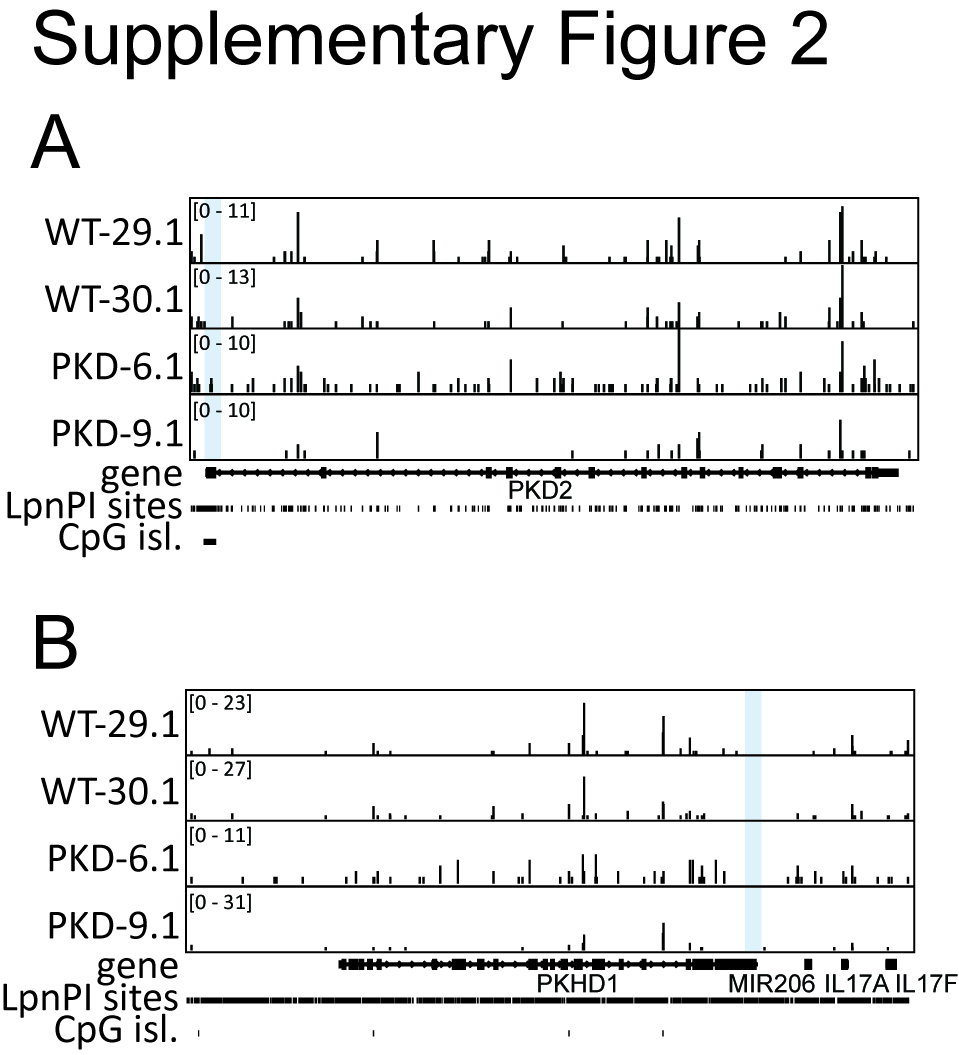

Supplement: Supplementary file 3 — Figure S2 MeD‐seq analysis of PKD2 and PKHD1 loci in WT and PKD TECs. MeD‐seq profiles of (A) PKD2, and (B) PKHD1, displaying read‐counts per LpnPI site, showing no gain in methylation in the promoter (blue), or other regions of both genes. [file SCT3-9-478-s003.tif]

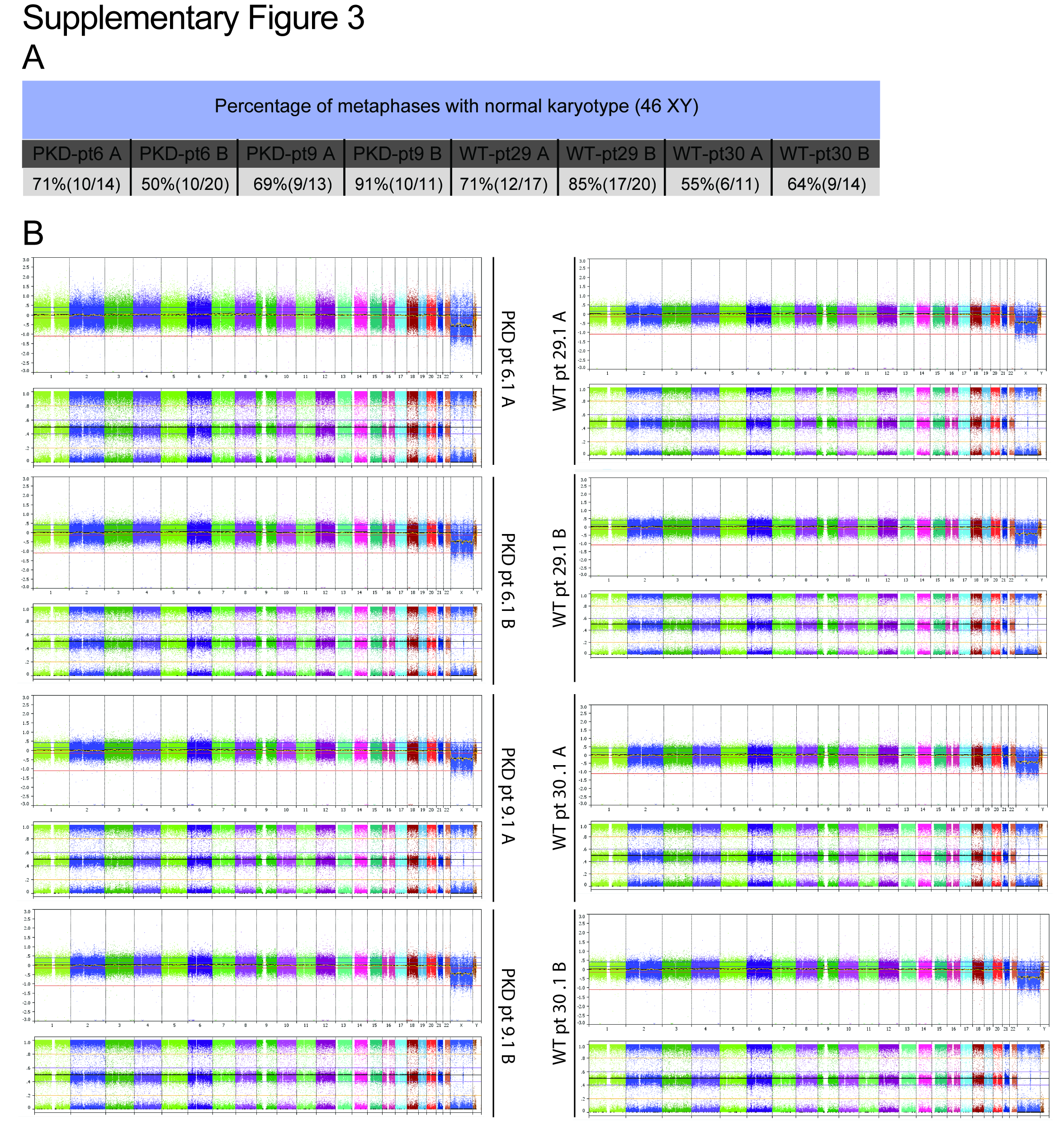

Supplement: Supplementary file 4 — Figure S3 Karyotype of iPSC lines. (A) Quantification of karyotyping of iPSC lines, cells with an abnormal karyotype only showed loss of chromosomes, with no preference for loss of one specific chromosome. (B) SNP Array analysis per clone showing Log(R ratio) potentially detecting gains and losses (top panels per clone), and loss of heterozygosity with B allele frequency (BAF) of 100% or 0% (bottom panels per clone). [file SCT3-9-478-s004.tif]

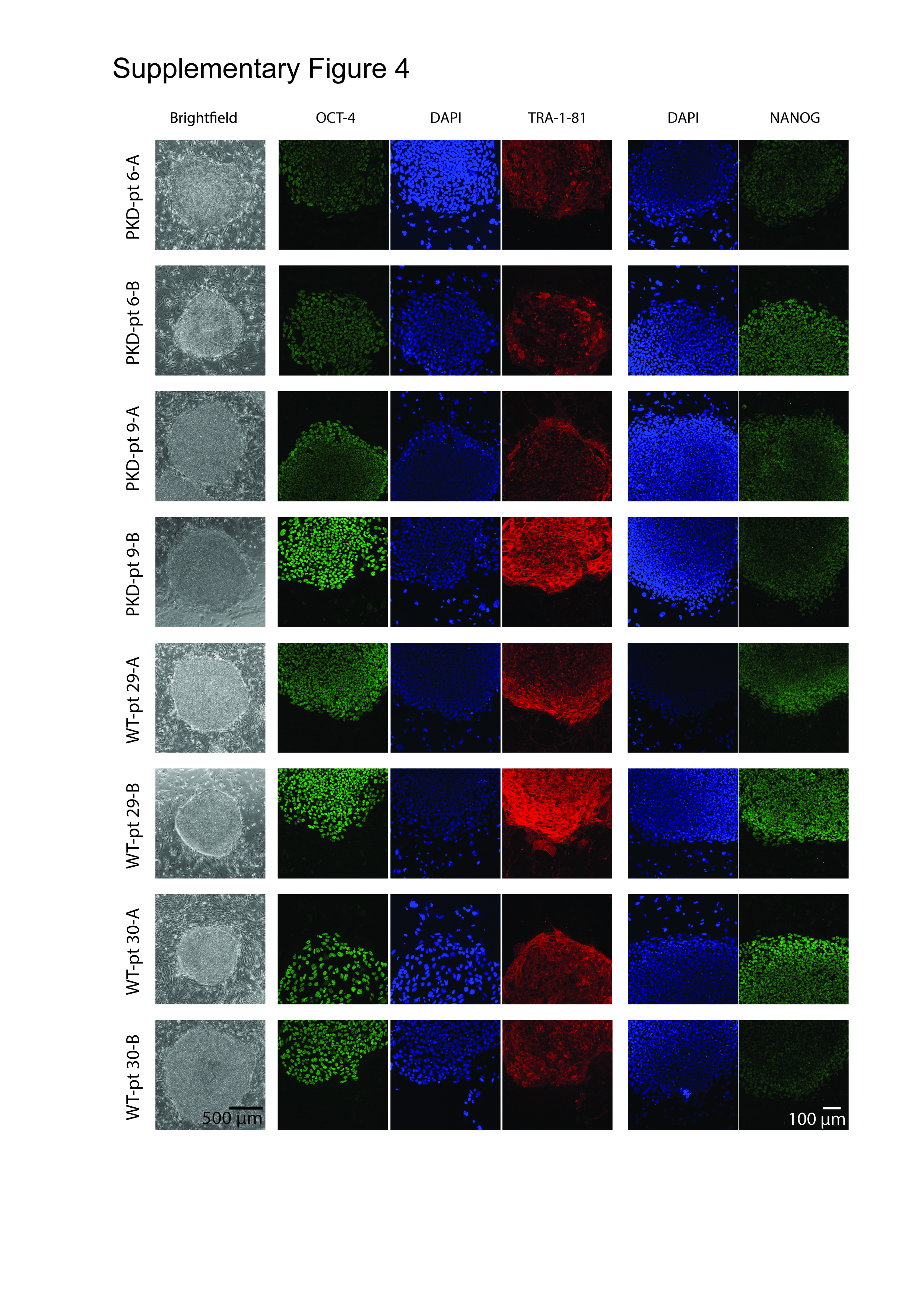

Supplement: Supplementary file 5 — Figure S4 Characterization of undifferentiated WT and PKD derived iPSC lines. Immuno‐fluorescence expression analysis of pluripotency markers OCT4 (FITC), TRA‐1‐81 (Rhodamine Red) and NANOG (FITC, DNA is DAPI/Blue) in iPSC lines derived from PKD and WT TEC cell lines. [file SCT3-9-478-s005.tif]

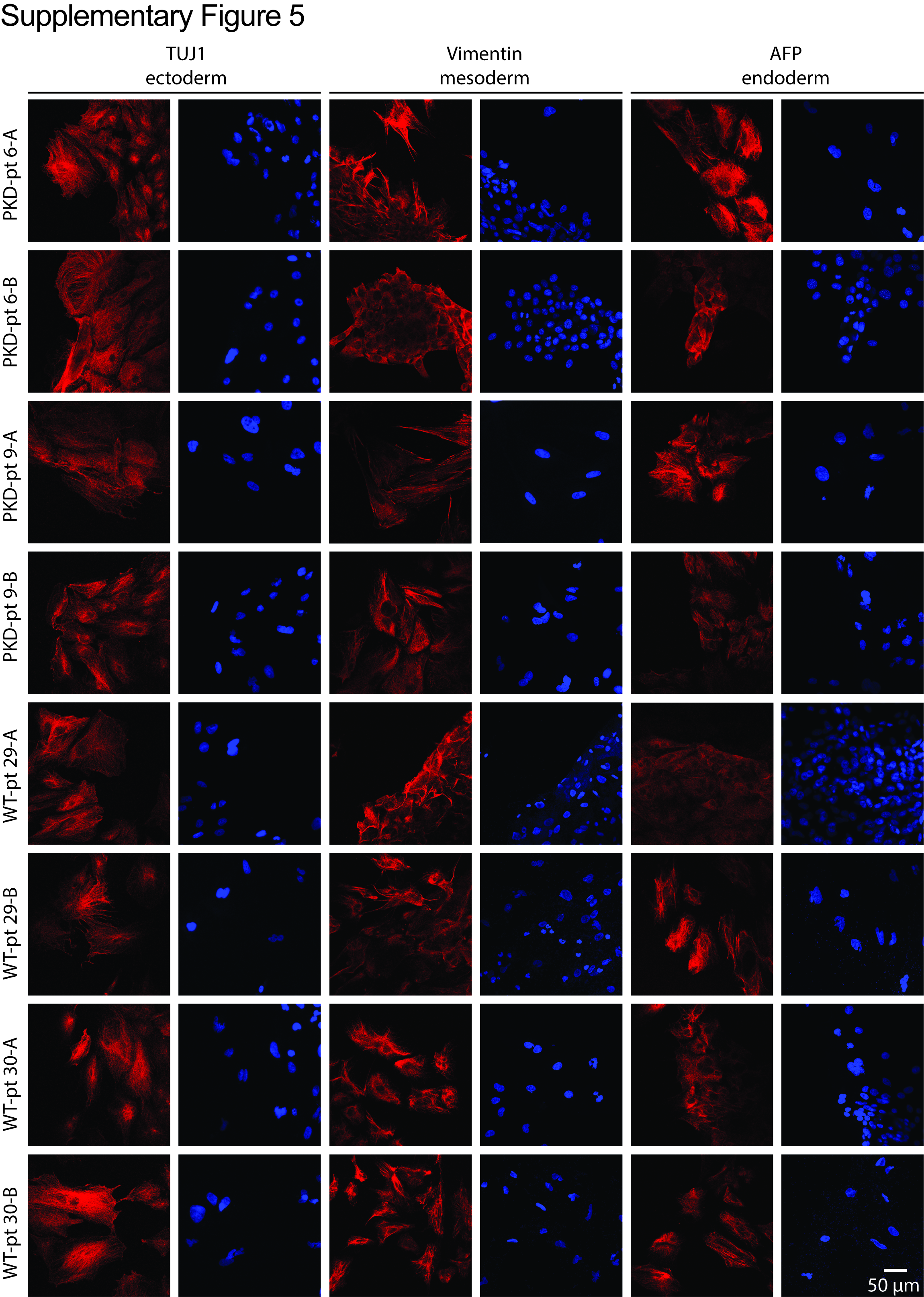

Supplement: Supplementary file 6 — Figure S5 EB differentiation and germ layer formation of PKD and WT iPSC lines. Immuno‐fluorescence expression analysis of endoderm, mesoderm and ectoderm markers AFP, Vimentin, and TUJ1 (Rhodamine red, DNA is DAPI/Blue) in EB differentiated iPSC lines derived from PKD and WT TEC cell lines (scale bar = 50 μm for all panels). [file SCT3-9-478-s006.tif]

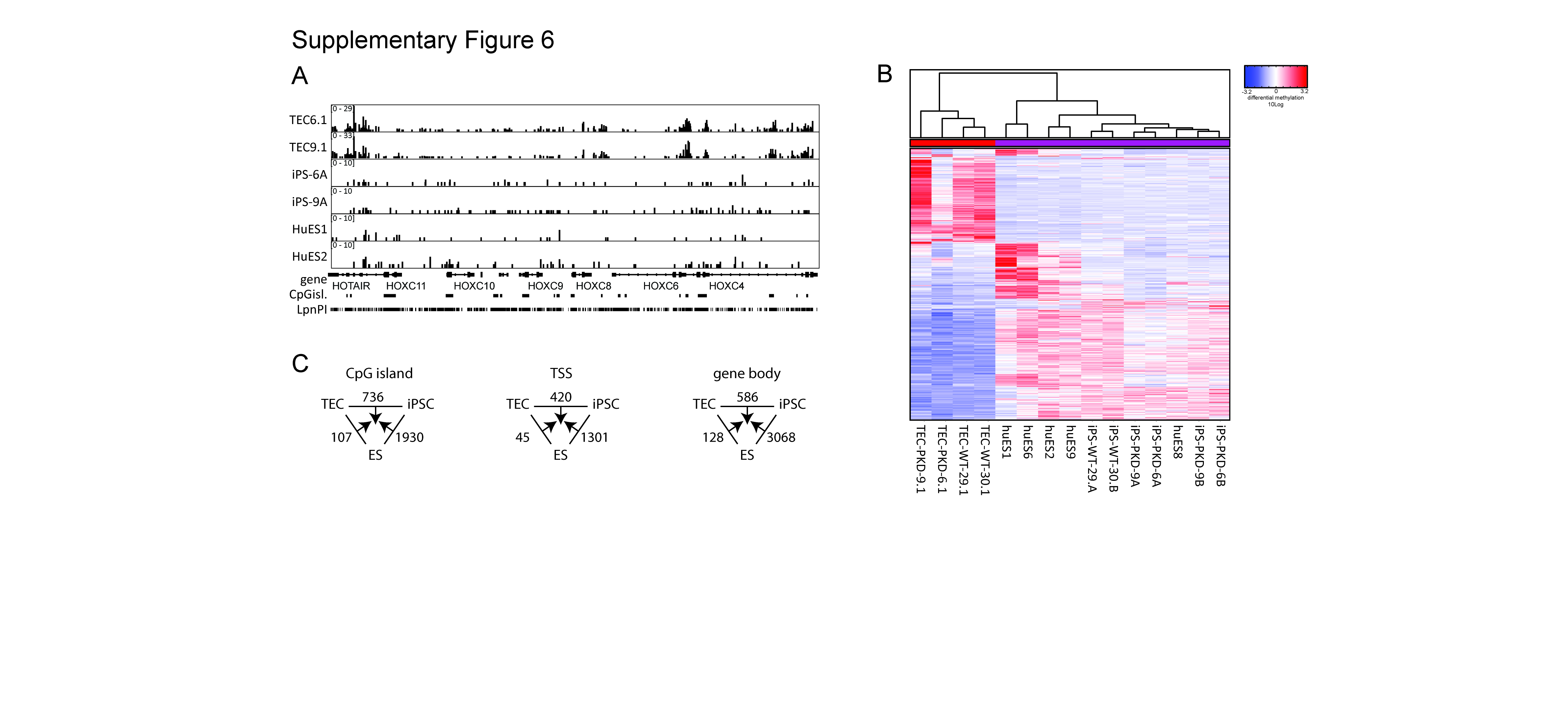

Supplement: Supplementary file 7 — Figure S6 Genes hypermethylated in gene body in PKD iPSCs. (A) MeD‐seq gene tracks of the HOXC locus in TEC, iPS and ES cell lines, showing loss of methylation in reprogramed iPS‐6A and iPS‐9A cell lines to a level similar to found in ESCs. (B) Unsupervised hierarchical clustering analysis of TEC, PKD iPS and control ES cell lines, based on TSS DMRs observed between inter cell line comparisons. (C) Overview of CpG island, TSS and gene body DMRs specific for TEC vs iPSC:ESC, iPSC vs ESC:TEC, and ESC vs TEC:iPSC comparisons. [file SCT3-9-478-s007.tif]
